# *Lactiplantibacillus plantarum*, *lactiplantibacillus pentosus* and inulin meal inclusion boost the metagenomic function of broiler chickens

**DOI:** 10.1186/s42523-023-00257-5

**Published:** 2023-08-03

**Authors:** Ilario Ferrocino, Ilaria Biasato, Sihem Dabbou, Elena Colombino, Kalliopi Rantsiou, Simone Squara, Marta Gariglio, Maria Teresa Capucchio, Laura Gasco, Chiara Emilia Cordero, Erica Liberto, Achille Schiavone, Luca Cocolin

**Affiliations:** 1 Department of Agricultural, Forest and Food Sciences, University of Turin, Turin, Italy; 2 Center Agriculture Food Environment (C3A), University of Trento, Turin, Italy; 3 Department of Veterinary Sciences, University of Turin, Turin, Italy; 4 Department of Drug Science and Technology, University of Turin, Turin, Italy

**Keywords:** Lactic acid bacteria, Probiotic, Prebiotic, Inulin, microbiota, Broiler chickens

## Abstract

**Background:**

The inclusion of alternative ingredients in poultry feed is foreseen to impact poultry gut microbiota. New feeding strategies (probiotics/prebiotics) must be adopted to allow sustainable productions. Therefore, the current study aimed to use metagenomics approaches to determine how dietary inclusion of prebiotic (inulin) plus a multi-strain probiotic mixture of *Lactiplantibacillus plantarum* and *Lactiplantibacillus pentosus* affected microbiota composition and functions of the gastro-intestinal tract of the broilers during production. Fecal samples were collected at the beginning of the trial and after 5, 11 and 32 days for metataxonomic analysis. At the end of the trial, broilers were submitted to anatomo-pathological investigations and caecal content was subjected to volatilome analysis and DNAseq.

**Results:**

Probiotic plus prebiotic inclusion did not significantly influence bird performance and did not produce histopathological alterations or changes in blood measurements, which indicates that the probiotic did not impair the overall health status of the birds. The multi-strain probiotic plus inulin inclusion in broilers increased the abundance of *Blautia*, *Faecalibacterium* and *Lachnospiraceae* and as a consequence an increased level of butyric acid was observed. In addition, the administration of probiotics plus inulin modified the gut microbiota composition also at strain level since probiotics alone or in combination with inulin select specific *Faecalibacterium prausnitzi* strain populations. The metagenomic analysis showed in probiotic plus prebiotic fed broilers a higher number of genes required for branched-chain amino acid biosynthesis belonging to selected *F. prausnitzi* strains, which are crucial in increasing immune function resistance to pathogens. In the presence of the probiotic/prebiotic a reduction in the occurrence of antibiotic resistance genes belonging to aminoglycoside, beta-lactamase and lincosamide family was observed.

**Conclusions:**

The positive microbiome modulation observed is particularly relevant, since the use of these alternative ingredients could promote a healthier status of the broiler’s gut.

**Supplementary Information:**

The online version contains supplementary material available at 10.1186/s42523-023-00257-5.

## Introduction

Broiler gastro-intestinal (GI) tract health is one of the key factors that must be considered to determine an improvement of poultry production systems, thus helping birds to properly develop by conferring resistance to external perturbations such as farming practices, climate changes, or occurrence of pathogens. A GI disbiosis can have a huge impact on animal weight gain and feed efficiency, impairing the absorption of nutrients, causing stress to the birds, and making animals more susceptible to be colonized by pathogens (e.g. *Salmonella*, *Campylobacter*) [1]. Use of antibiotics in farming procedures for disease prevention and growth promotion under crowded conditions can reduce the microbial diversity of the GI tract with issues including malabsorption, and can increase the resistome of the broilers [2]. The subsequent spread of antimicrobial resistance genes (ARGs) from animals to the environment can potentially compromise human health as recognized by the One Health concept [3]. Several studies have shown that the use of antibiotics in farms will lead to an increased occurrence of ARGs, and that a reduction of their usage will eventually reduce it [2]. In this light, a healthy gut microbiome of broiler chickens can help in immunomodulation as well as conferring resistance to colonization by pathogens and, as a consequence, can reduce the use of antibiotics. Several strategies are currently proposed to improve gut health and contrast the occurrence of pathogens including: administration of dietary prebiotics [4–6], post-biotics [7], acidifiers or plant extracts [8], and probiotics. Among the latter, those currently used in poultry farming belong to the genus *Lactobacillus*, *Bacillus*, *Bifidobacterium*, *Pediococcus*, *Streptococcus*, *Enterococcus* and *Saccharomyces* [1, 9]. Probiotics preparation with lactic acid bacteria (LAB) can have a direct or indirect influence on animal welfare, reduce oxidative stress [1], promote growth performance, improve meat quality and fat deposition [10], improve blood parameters related to immunity [11], and confer resistance to pathogens infection [4, 12, 13]. However, numerous studies have focused on the positive effects of probiotics in animals, but the mechanisms by which probiotics can successfully exert beneficial effects remain unclear [10], even because in some studies LABs probiotic strains did not remain in the GIT long-term [14]. Apart from the use of LABs probiotics, prebiotics (including fibers or indigestible carbohydrates) are often used as a dietary supplement to increase broiler chickens performance and reduce pathogens [5, 7]. Since dietary prebiotics pass through the proximal portion of the GI tract, they have the ability to interact with intestinal microbiota [4]. Among prebiotics, inulin is an alternative to antibiotic growth promoters in chicken, since at lower level of inclusion positive effects on body weight gain, feed intake, food conversion rate and biochemical parameters were observed [15]. Inulin is a mixture of oligomers and polymers that occurs naturally in many plants, especially chicory. Literature showed that depending on the inclusion percentage, results are conflicting. The 10% w/w of inclusion in the normal diet showed the potential to beneficially impact broiler performance and promote gut health via microbial fermentation [16]. Inulin can modulate the GI microbiome by affecting short-chain fatty acids (SCFAs) metabolism and microbial functional profiles [17]. The mechanism of action of inulin is rather complex, since is selectively fermented into free fructose available for microbial development. Thus, depending on the microbiota composition found in the GI, its effect can vary [18].

Therefore, the current study aimed to use a multi-level approach based on intestinal microscopic features,metagenomics and metabolomics approaches to determine how dietary inclusion of prebiotic (inulin) plus a multi-strain probiotic mixture of *Lactiplantibacillus plantarum* and *Lactiplantibacillus pentosus* affected microbiota composition and functions of the GI tract of the broiler chickens. Poultry food chain suffers for old and long-lasting problems related to food safety issues (i.e., foodborne pathogens and antibiotic resistance), particularly when intensive breeding is taken into consideration. New feeding strategies (probiotics/prebiotics) must be adopted to allow sustainable productions, and to reduce pathogens and antibiotic resistance in the gastrointestinal tract, thus resulting in an improvement of animal welfare and the safety and quality of poultry meat. The application of multidisciplinary approach can help investigate how this new ingredient can modulate the gut broiler microbiome.

## Results and discussions

**Growth performance and gut morphology** All birds remained healthy throughout the trial and no mortality was recorded. No significant differences were observed for growth performance (live weight and feed conversion ratio for both considered period 1–11 d and 12–32 d) as a function of the supplementation (Table 1).

**Table 1:** Growth performances. Histopathological findings and blood metabolites of broiler chickens

	C	LABs	I	MIX	SEM	*P*-value
**LW at 1 d. g**	43.17	43.54	43.65	43.30	0.65	0.99
**LW at 11 d. g**	299.18	310.00	302.70	310.43	4.78	0.81
**LW at 33 d. g**	1798.67	1838.80	1764.22	1867.00	42.77	0.37
**FCR 1–11 d**	1.143	1.109	1.117	1.122	0.04	0.54
**FCR 12–32 d**	1.269	1.030	1.314	1.219	0.11	0.67
**Spleen**	0.37	0.57	0.25	0.25	0.09	0.54
**Liver**	0.19	0.12	0.62	0.31	0.09	0.16
**Thymus**	Absence of alterations					
**Bursa of Fabricius**	1.31	1.25	1.5	1.31	0.07	0.59
**Glandular stomach**	2.25	1.88	1.38	1.63	0.16	0.24
**Gut**	3.00	2.75	2.38	2.00	0.23	0.25
**Albumine (g/dL)**	1.02	1.18	1.08	1.12	0.03	0.25
**Alanine aminotransferase (UI/L)**	2.75	2.25	2.42	2.50	0.25	0.78
**Aspartate aminotransferase (UI/L)**	344.33	323.92	267.50	265.67	15.78	0.16
**Total Cholesterol (mg/dL)**	88.58	110.33	111.33	109.83	3.81	0.08
**Triglycerides (mg/dL)**	26.92	37.92	31.67	47.67	4.63	0.71
**Uric acid (mg/dL)**	3.73	4.45	2.69	4.73	0.37	0.21
**Cholesterol HDL (mg/dL)**	71.50	85.75	86.33	85.33	3.29	0.08
**Cholesterol LDL (mg/dL)**	17.08	24.58	25.00	24.50	1.28	0.09
**Creatinine (mg/dL)**	0.08	0.06	0.05	0.08	0.01	0.30
**Total Protein (g/dL)**	3.48	3.75	3.56	3.62	0.06	0.57
**Phosphorous (mg/dl)**	89.37	95.98	70.22	95.20	11.33	0.86
**Chlorine (mmol/L)**	125.90	128.27	125.32	129.10	1.13	0.62
**Potassium (mmol/L)**	5.27	5.06	4.74	5.80	0.26	0.48
**Magnesium (mEq/l)**	6.02	5.62	5.53	5.93	0.16	0.57
**Iron (µg/dl)**	85.67	110.17	104.00	113.17	6.22	0.56
**Alkaline Phosphatase (U/L)**	9312.00	9024.00	7790.00	7052.67	672.07	0.69
**Calcium (mg/dl)**	42.68	43.32	41.15	42.82	0.87	0.67
**Sodium (mmol/L)**	177.90	179.52	174.87	182.08	1.69	0.39

C = control; LABs *= Lactiplantibacillus plantarum + L. pentosus*; I = inulin; MIX = inulin + *Lactiplantibacillus plantarum + L. pentosus Lactobacillus;* LW = live Weight (g); ADG = Average Daily Gain (g/d); ADFI = Average Daily Feed Intake (g/d); FCR = Feed Conversion Ratio; SEM: pooled standard error of the mean

The effects of dietary treatment, gut segment and interaction between diet and gut segment on the gut morphometric indices of the broiler chickens are summarized in Additional file 1: Tables S1 and S2. In details, only intestinal segment and interaction between diet and intestinal segment significantly affected the morphometric indices (*P* < 0.05). On the contrary, there was no significant influence of diet (*P* > 0.05, Additional file 1: Table S2). In particular, the jejunum of LABs-fed broilers displayed longer villi (P = 0.005) than I and MIX groups (2.33 mm vs. 1.64 and 1.78, respectively), but analogous to the C birds (2.14 mm). Similarly, greater Vh/Cd (P < 0.001) were identified in the jejunum of LABs broilers (12.33) when compared to the I and MIX birds (8.51 and 9.77, respectively), still being analogous to that observed in the C group (10.89). Despite changes being identified in jejunum only, the herein highlighted scenario represents a positive outcome, as jejunum represents a major site of nutrient digestion and absorption in poultry [19]. In particular, the supplementation of LABs seems to be slightly preferable for preserving an effective luminal absorptive area and, in turn, nutrient absorption – of which Vh and Vh/Cd are useful indicators [20]. Independently of the dietary treatment, duodenum showed greater Vh and Vh/Cd (*P* < 0.001) than the other gut segments, with morphometric indices being also greater (*P* < 0.001) in jejunum when compared to the ileum. Furthermore, higher Cd (*P* < 0.05) was observed in duodenum than ileum (Additional file 1: Table S2). The identification of a proximodistal decreasing gradient of the morphometric indices from the duodenum to the ileum reflects the physiological development of the bird small intestine [19]. However, our results are overall not surprising, since the daily administration of a multi-strain probiotic [21], the combination of inulin plus post-biotic [7] or inulin alone [5] in broilers have previously been reported to not affect growth performance and morphology.

### Histopathological findings

Mild histopathological alterations developed in all the intestinal tracts, glandular stomach (proventriculus), spleen, liver and bursa of Fabricius for all the dietary treatments, regardless of the treatment. Occasionally, lymphoplasmacytic infiltrates and lymphoid tissue hyperplasia were observed in glandular stomach (proventriculus), duodenum, jejunum and ileum. Slight lymphoid tissue hyperplasia was detected in caecum. The spleen showed white pulp hyperplasia. Steatosis or vacuolar degeneration of the hepatocytes, as well as lymphoid tissue activation, was observed in liver. Bursa of Fabricius showed follicular depletion. However, dietary treatments did not affect the severity of the observed histopathological alterations in any of the organs (P > 0.05, Table 1), and the alterations varied from absent to mild in all the animals. Other studies reported that a multi-strain administration of probiotic did not alter the histopathological features of broilers, even if probiotic administration could determine an increase in the weight of the bursa of Fabricius, which may indicate an improvement in the bird immune system [21]. Moreover, different probiotic strains exhibit different properties and clinical effects that are strain dependent, and the combination of strains may have an effect on the efficacy of a multi-strain probiotic [21].

### Metabolomic features of broiler

**Fig. 1:**
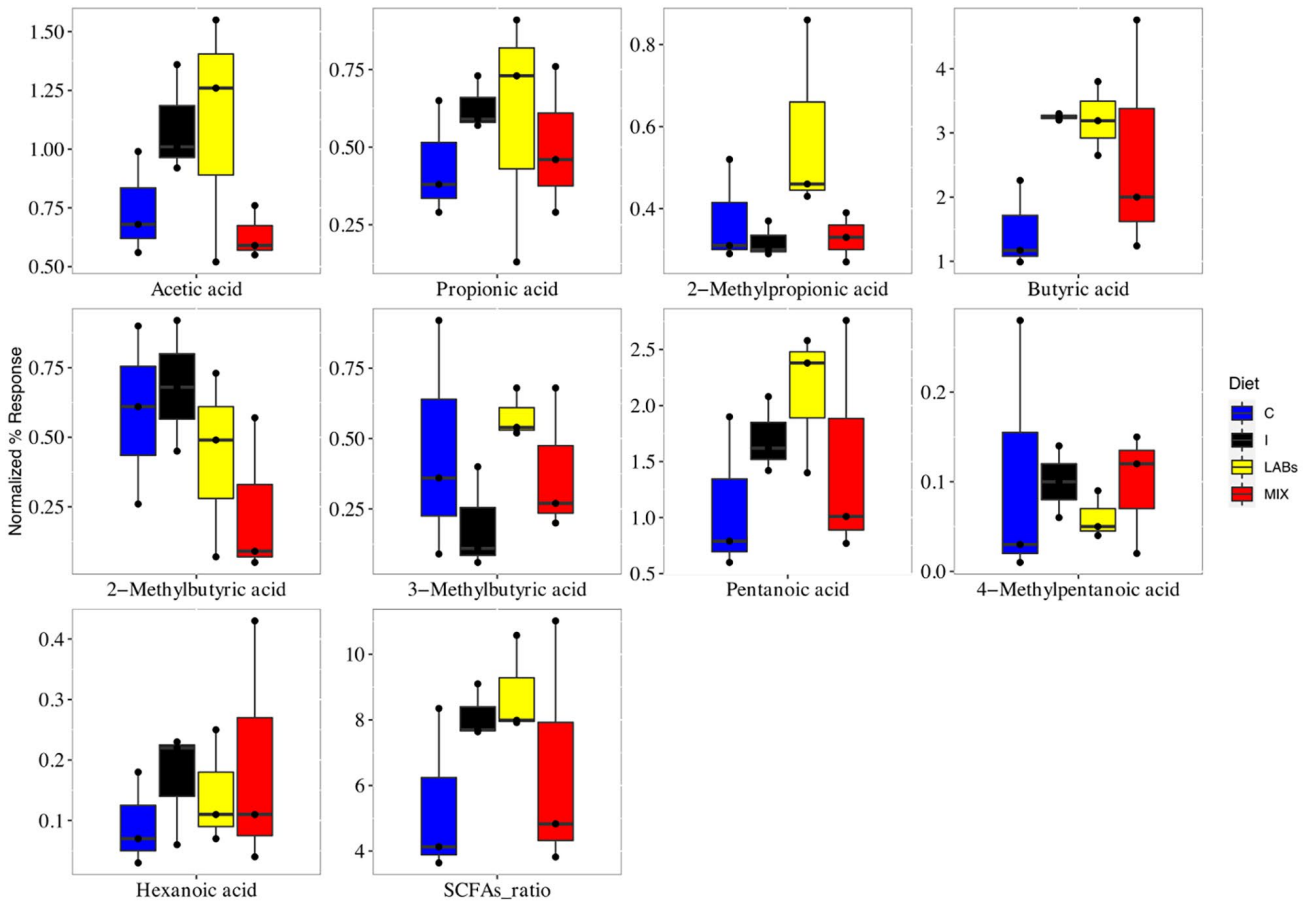
Short chain fatty acid composition of broiler gut. Boxplots of the SCFAs detected in the cecal samples in broilers fed with control (C), inulin (I), Inulin plus probiotics (MIX) and probiotics (LABs).

Blood measurements of the birds were not affected by the dietary treatment (P > 0.05; Table 1). Gut volatilome is influenced by several factors, and the microbiota composition has a primary role, thereby its fingerprint does not suggest metabolic changes due to the stability of the microbiome. The acid volatile fractions detected by GC×GC-TOF-MS in cecal samples are shown in Fig. 1.

We observed that only butyricbutyric acid was mostly produced by broilers fed LABs (P < 0.05), while the other SCFAs as well as the total SCFAs ratio were not significant different among the experimental diets when compared to the C group. This is due to the probiotics used in this study that was selected due to its ability to produce butyric acid [22]. However also the natural anaerobic microbiota (e.g. *Clostridium*, *Bacteroides* and *Propionibacterium*) of the cecal samples can also produce propionic and butyric acid influencing the SCFAs profile.

The positive effects of butyric acid are well-known in broilers, it increases intestinal mucin production and improves tight-junction integrity [12]. In addition, we observed that the SCFAs profile was overall not affected by the presence of inulin as already proven in human studies [23]. Inulin alone does not have a strong effect on growth performance [16], but in combination with probiotic strains it synergistically stimulates intestinal epithelial cells, immune cells and gut microbes [15, 18]. Microbial cross-feeding has a huge impact on the final balance of SCFAs, and several beneficial microbes (including *Faecalibacterium prausnitzii*) showed the ability to use SCFAs as a source of energy or shows a strong requirement to growth [24, 25]. Lack of difference in SCFAs among the experimental treatments can be explain by this cross-fed mechanism.

### Microbiota characterization of broilers with dietary probiotic inclusion

A total of 65 fecal samples were analyzed throughout the experiment [time 0 (5 samples), and after 5 (20 samples), 11 (20 samples) and 32 days (20 samples)]. After amplicon-based sequencing, the rarefaction analysis and the estimated sample coverage indicated that there was a satisfactory coverage of all the samples (ESC median value of 96.52%). Regarding alpha-diversity value, only Shannon index increased as a function of time (*P* = 0.011) due to the increased size of the broilers [26], while probiotic inclusion did not modify the diversity within each sample (data not shown).

**Fig. 2:**
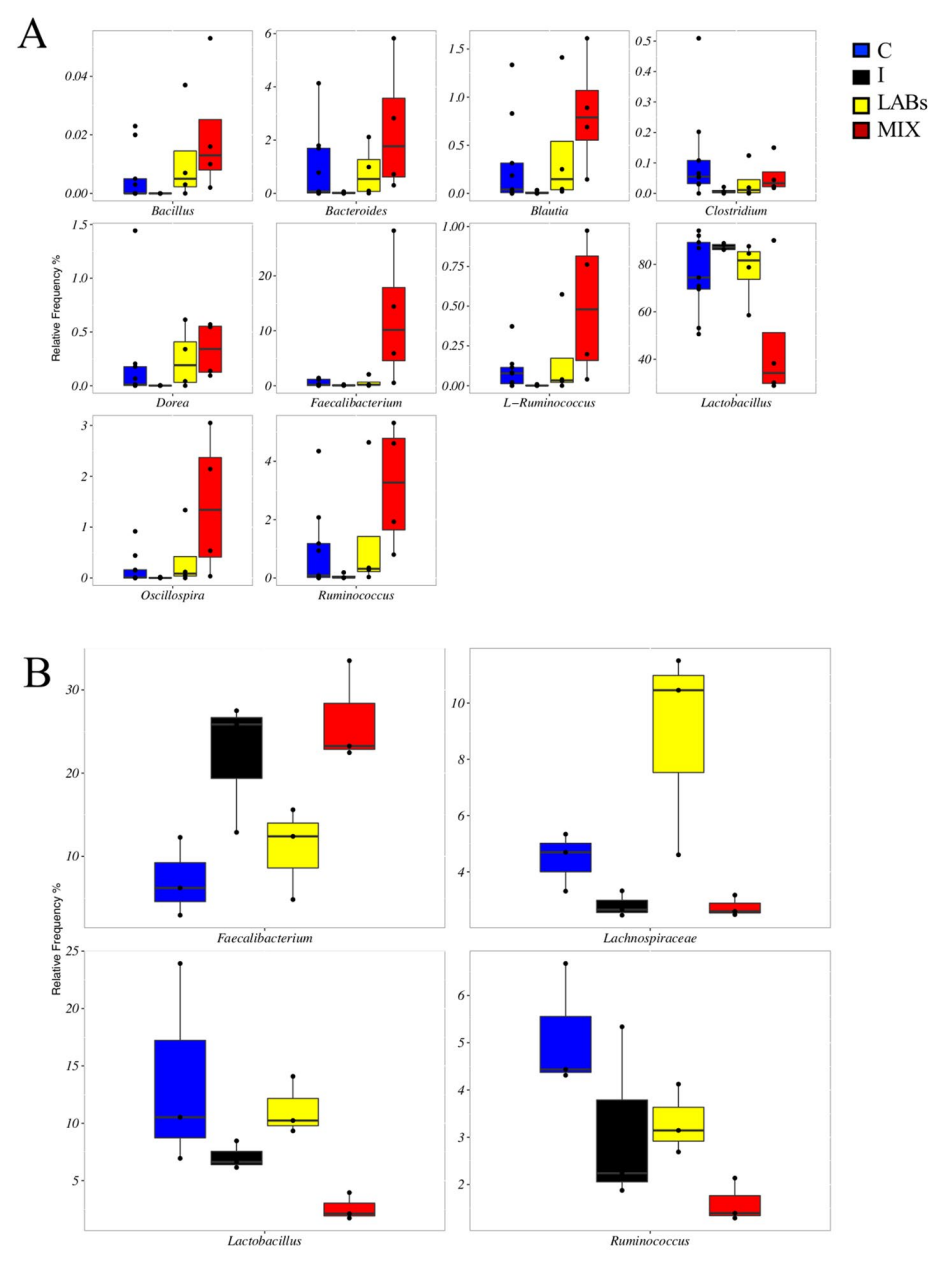
Metataxonomic analysis of broiler gut. Boxplots of the ASVs significantly different based on Wilcoxon test (FDR < 0.05) between broilers fed with control (C), inulin (I), Inulin plus probiotics (MIX) and probiotics (LABs) in faecal samples at the end of the trial (Plot A) and in the cecal samples (Plot B)

We observed that the factor “sampling Time” was the one that modified the microbiota composition (Additional file 1: Table S3). Dietary supplement showed a marginal effect on *Sutterella* (P = 0.036) and *Turicibacter* (*P* = 0.043) only. However, a specific signature of fecal samples at 32 days was observed for the different dietary treatments. Broilers fed MIX showed an increase in the fecal frequency of *Bacillus*, *Blautia*, *Dorea*, *Faecalibacterium*, L − *Ruminococcus, Oscillospira* and R-*Ruminococcus*, while ASVs belonging to *Lactobacillus* decreased (FDR < 0.05 Fig. 2A). Amplicon based sequencing of cecal microbiota showed few differences: in particular, *Faecalibacterium* was mainly associated with MIX – while *Ruminococcus* decreased – and *Lachnospiraceae* and *Lactobacillus* were mainly associated with LABs-fed broilers (FDR < 0.05, Fig. 2B).

Consistently with these results, the increased presence of the SCFAs-producing bacteria such as *Faecalibacterium*, *Blautia, Dorea* and *Ruminococcus* are related to gut health in the older chicks and are linked to high chicken productivity [26]. It has already been shown in dog models that lactic acid bacteria as a probiotic supplementation help in the development of these taxa [27]. Here we observed that our probiotic mixture led to the enrichment of these beneficial microbes, as observed for other lactic acid bacteria in broilers [28]. These taxa are in a homeostatic balance with the host and can guarantee the protection of the gut against pathogens due to the production of antimicrobial compounds or by the competition for nutrients [29]. The presence of inulin helps in maintaining gut homeostasis [30]. This fructooligosaccharide (FOS) stimulates the growth of beneficial *Faecalibacterium* since it is metabolized by colonic microbiota, mainly by lactobacilli, to oligosaccharides and monosaccharides and then fermented to SCFAs that serve as a source of energy for gut commensals [31–33]. Inulin degradation mediated by *Bifidobacterium* and LABs resulted in release of free fructose, lactate and acetate that are easily accessible substrates for colonic microbes [34], in particular *Faecalibacterium* that needs acetate as a source of energy.

### Metagenome assembled genomes (MAGs)

**Fig. 3:**
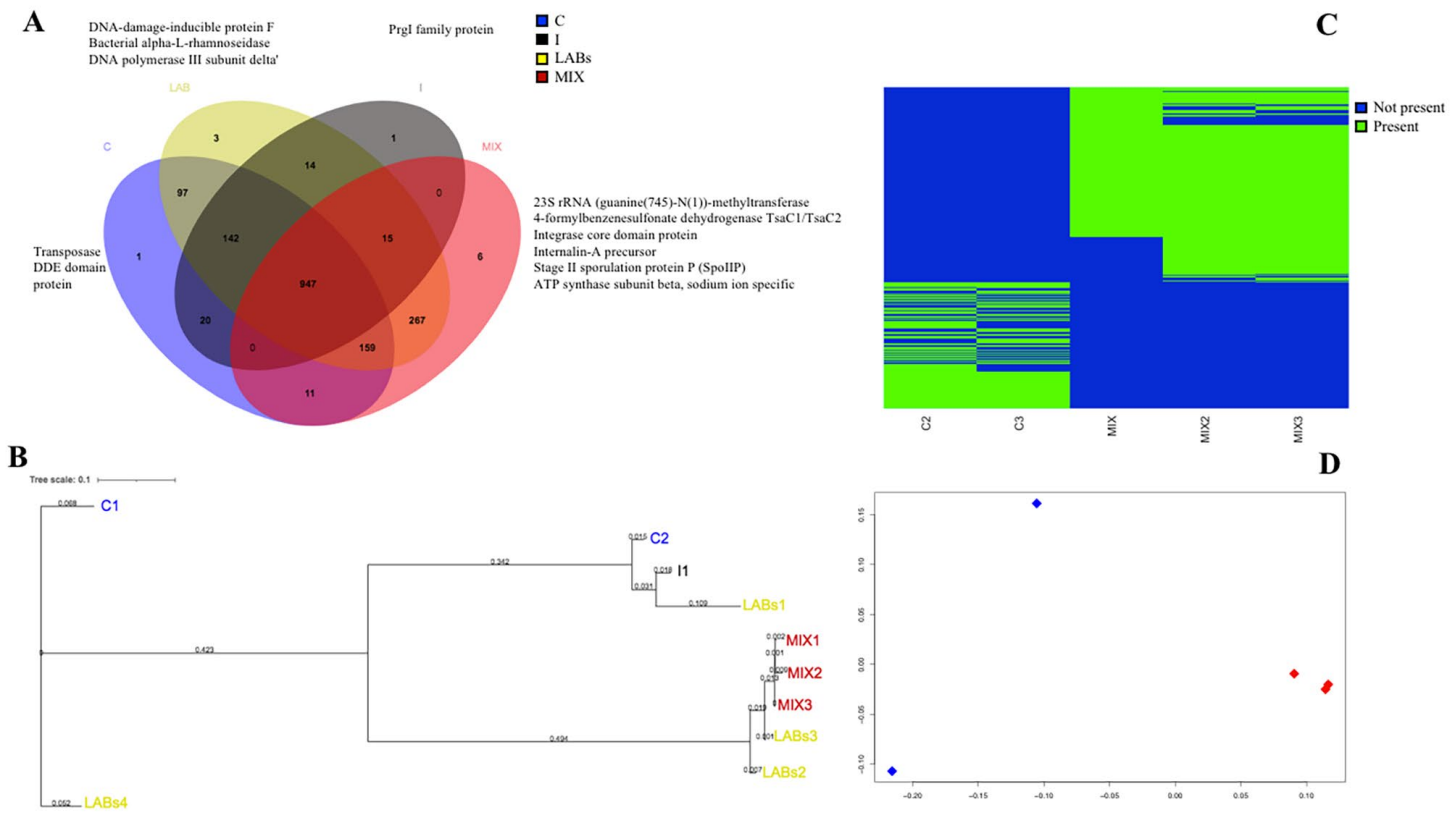
*Faecalibacterium prausnitzii* pattern in broiler metagenomes. **(A)** Venn diagrams depicting the overlap between genes common or unique between the four feeding strategies control (C), inulin (I), Inulin plus probiotics (MIX) and probiotics (LABs). **(B)** Phylogenetic tree built on concatenated *-F. prausnitzii* genes extracted from assembled metagenomes. **(C)** Presence-absence of the *F. prausnitzii* MAGs genes. **(D)** Principal coordinates analysis based on *F. prausnitzii* pangenome related to control and Inulin plus probiotics (MIX) samples

Microbial communities profiling from metagenomic shotgun data is helping in estimate relative abundance without the bias of an amplification step and therefore potentially can be more accurate in microbial quantification as well as allowing species-level resolution. By this approach, we observed that several lactic acid bacteria were present in the cecal content of the broilers (among them: *Lactobacillus gallinarum*, *Lactobacillus crispatus, Limosilactobacillus reuteri, Ligilactobacillus aviarius*). Further, from the metagenomic data the presence of *Lactiplantibacillus pentosus* (species included in the probiotic supplement), in LABs and MIX samples, was revealed (data not shown). We then searched by metagenomic binning the presence of *Lactiplantibacillus plantarum* genomes, we were not able to find their sequence in the metagenome. Persistence of *Lactobacillus* has been shown to be a transient feature and only some species (such as *Lactobacillus mucosae*) are able to stably colonize the GI, while other species did not colonize the ileum [35]. We can suppose that *Lactiplantibacillus plantarum* was not able to colonize the GI in a stable way in our broilers since its differences in persistence may in part be explained by the different animal models used [36]. However, the administration of probiotics plus inulin modified the gut microbiota composition also at strain level. Several high-quality MAGs (52) were identified in the metagenomes dataset belonging to: *Alistipes inops* (5), *Alistipes* sp (6), *Anaerostipes* sp (1), *Bacteroides uniformis* (9), *Blautia* SGB4801 (2), *Faecalibacterium prausnitz*i (10), *Flavonifractor* sp (1), *Helicobacter pullorum* (8), *Lachnoclostridium* sp (4), *Parabacteroides distasonis* (4) and *Pseudoflavonifractor* sp (1). MAGs belonging to *Faecalibacterium prausnitzi* were furtherly characterized at high-resolution phylogeny with PhyloPhlAn3. Phylogenetic trees showed that *Faecalibacterium prausnitzi* MAGs from LABs and MIX broilers had similar potential function, while the ones from C samples belonged to a different cluster (Fig. 3).

Distribution of the MAGs was found to be related to different diets, thus highlighting a selection process at the strain level as already observed in humans [37]. Probiotics alone or in combination with inulin apparently select specific *Faecalibacterium prausnitzi* strain populations. We then observed a potential functional diversity across the different MAGs. Venn diagram (Fig. 3) depicted the overlap between *Faecalibacterium prausnitzi* genes common or unique to the different dietary regimes. Among 1683 genes, few genes were unique to each dietary treatment (belonging to genetic information processing and replication and repair orthology). When comparing MAGs from C vs. MIX, a total of 1117 genes were shared, while 288 where unique to MAGs from MIX and 260 to MAGs from C (Fig. 3). *Faecalibacterium* pangenome of MIX broilers displayed the prevalence of genes involved in carbohydrate metabolism and degradation (such as beta-glucosidase, carbohydrate acetyl esterase/feruloyl esterase, acetate kinase and cyclomaltodextrinase), genes coding for branched-chain amino acids biosynthesis (methionine aminotransferase) and phosphotransferase system (PTS) transport of fructose, as well as genes encoding for antibiotic resistance (Beta-lactamase CARB-6 precursor and chloramphenicol acetyltransferase). The presence of acetate kinase confirms the ability of the strains present in MIX broiler to use SCFAs a source energy. On the other hand, *Faecalibacterium* pangenome of C broilers displayed the prevalence of genes involved in aminoacid biosynthesis and transport (arginine, serine and ornithine), fatty acid biosynthesis, and several genes encoding for antibiotic resistance (beta-lactamase, penicillin and multidrug resistance genes). Free fructose produced by Lactobacilli from inulin [34] increase the number of certain *Faecalibacterium* strains [38]. In vitro study showed that only some members of *F. prausnitzii* population are selectively stimulated by inulin as a carbon source, since fructose is a preferred one. Pangenome analysis of *F. prausnitzii* from MIX broilers showed the predominance of genes involved in the transport of fructose, thus we can speculate that *F. prausnitzii* populations were selected under the synergic effect of prebiotic/probiotic intakes [33, 39].

### Shift in metagenomic function due to probiotic plus inulin

**Fig. 4:**
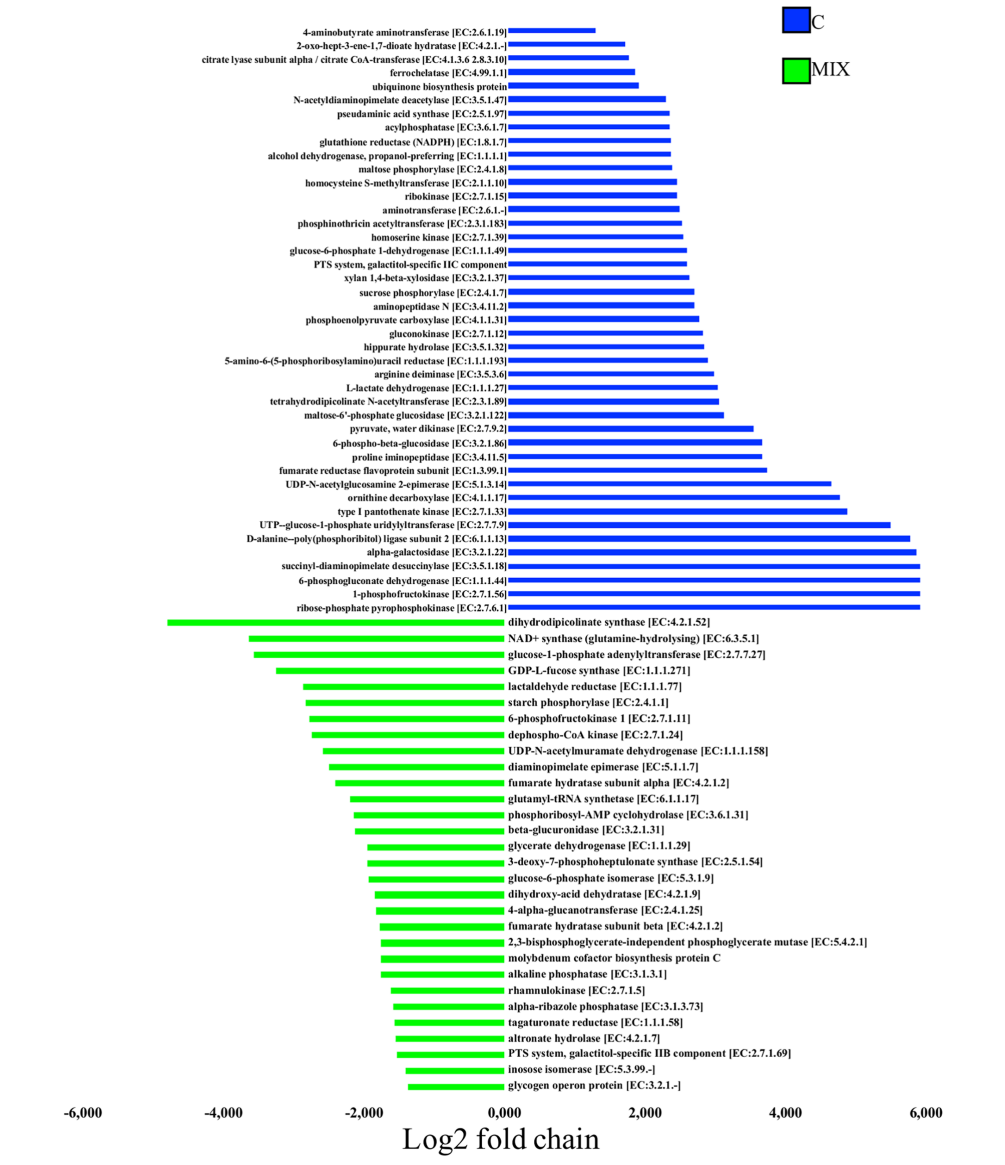
KEGG genes differentially abundant in broiler gut. The plot shows the log2-fold changes for KEGG genes belonging to Aminoacid and Carbohydrate metabolism statistically significant (FDR < 0.05) between broilers fed with control (C) and Inulin plus probiotics (MIX) using the Benjamini-Hochberg adjustment as implemented in DESeq2. Positive log 2 fold changes indicate genes most abundant in C samples, Negative log 2 fold changes indicate genes most abundant in MIX samples

More than 1128 KEGG genes were identified in the datasets, but few differences only were observed by comparing control samples, C vs. inulin (Additional file 2: Table S4) and control samples vs. LABs broilers (Additional file 2: Table S5). When comparing control vs. MIX, several genes were found to be most abundant in MIX samples (FDR < 0.05, Additional file 2: Table S6 and Fig. 4).

In details, the metagenome potential function of MIX broilers showed a highest number of KEGG genes involved in amino acid metabolism and carbohydrate metabolism (FDR < 0.05). In particular, we observed the highest presence of dihydroxy-acid dehydratase [EC:4.2.1.9] (DHAD), a key gene crucial for branched-chain amino acid (BCAA) biosynthesis [40] belonging to *Faecalibacterium*, from MIX broilers, as well as a high presence of BCAA transport genes. An increasing level of BCAA increases resistance to birds to external factors like exposure to mycotoxins [41]. We also observed a significant increase in dihydrodipicolinate synthase [EC:4.2.1.52] and Diaminopimelate epimerase (DapF) [EC:5.1.1.7], which are two KEGG genes that catalyze the first and the last step in the biosynthesis of lysine belonging to *Faecalibacterium* from MIX broilers. It is shown that an increase in lysine can reduce the amount of fat in the carcass and promote the muscle mass [42]. A lower level of lysine can reduce antibody response and cell-mediated immunity in broilers [43].

On the other hand, a reduction in ornithine decarboxylase and arginine deiminase KEGG genes responsible for the synthesis of putrescine and ornithine was observed in MIX- and Inulin-fed broilers when compared to the C (FDR < 0.05, Additional file 2: Table S6 and Fig. 4). This biogenic amine from one side can help promote the growth of birds (although its role is controversial), but an excess can also be considered a negative factor [44, 45]. Broilers fed MIX were enriched in dihydroxy-acid dehydratase [DHAD, EC:4.2.1.9], *O-*acetyltransferase [EC:2.3.1.30] and glutamyl-tRNA synthetase [EC:6.1.1.17]. The DHAD is involved in the immune response against pathogens [40], while O-acetyltransferase and glutamyl-tRNA synthetase involved in the synthesis of cysteine from serine. Cysteine degradation by the microbiota emerged as a dominant pathway for H_2_S production. Lower amount of H_2_S reduce mucosal inflammation and maintains the integrity of the mucus layer [46, 47]. In addition, a higher level of cysteine can suppress pro-inflammatory genes and is known as an antioxidant exerting beneficial immunological effects [48]. Regarding carbohydrate metabolism, n increase in the presence of lactaldehyde reductase [EC:1.1.1.77] and GDP-L-fucose synthase [EC:1.1.1.271] belonging to *Faecalibacterium* from MIX broilers, a key gene in the production of propionate via propanediol pathway [49], was also observed. A tendency of an increased level of propionic acid was here observed from the volatilome, even if not statistically significant. We then observed a high presence of inosose isomerase that confers the ability to convert chiro-inositol to scyllo-inosose, an intermediate of myo-inositol fermentation [50] that belongs to B group vitamins, involved in endocrine modulation and potentially responsible for increased growth rate [51]. In addition, the metagenome of broilers fed MIX showed the highest abundance of glucose-1-phosphate adenylyltransferase [EC:2.7.7.27] – potentially involved in energy storage through glycogen synthesis [52] – and alkaline phosphatase [EC:3.1.3.1], both belonging to *Faecalibacterium*. Alkaline phosphatase is known to maintain normal gut microbial homeostasis, since it decreases lipopolysaccharide production and gut permeability, and, ultimately, reduces metabolic endotoxemia and inflammation [53].

### Resistome Profile

**Fig. 5:**
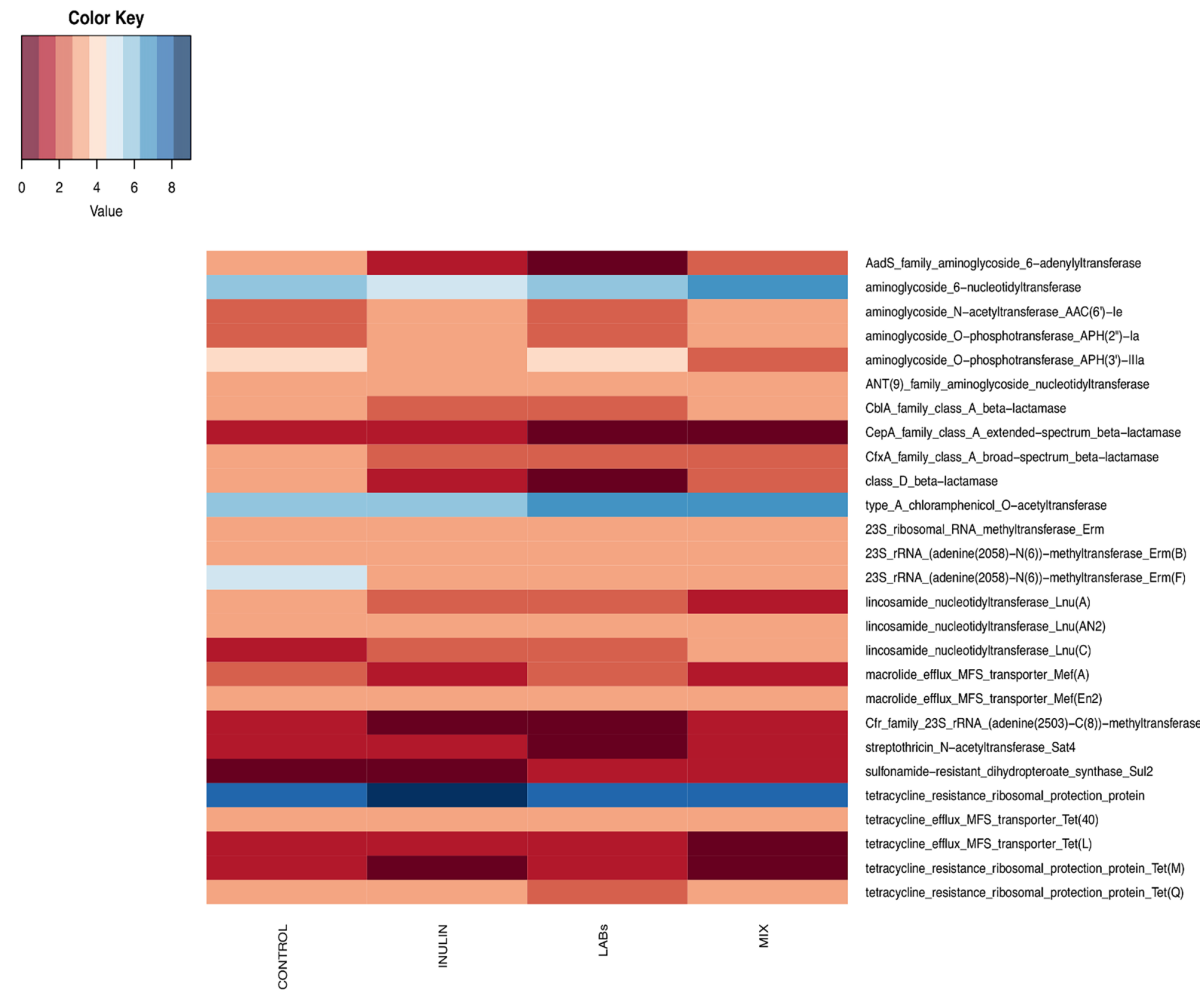
Antibiotic resistance genes distribution in broiler gut. Heatmap showing distribution of the Antibiotic resistance genes (ARGs) detected in broilers fed with control (C), inulin (I), Inulin plus probiotics (MIX) and probiotics (LABs). Colors represent the frequency of the ARGs from low frequency (dark red) to highest frequency (dark blue)

Antimicrobial resistance genes (ARGs) are one of the major public health problems due to the horizontal gene transfer between species in the gut and the spread in the environment. Here we used *AMRFinder*, a highly accurate detection system to check the presence of AMR genes in metagenome [54]. A variety of ARGs (280) conferring resistance to various antibiotics such as aminoglycoside (26%), tetracycline (21%), erythromycin (13%), beta-lactam (10%), lincosamide (10%) and macrolide (6%) were identified in the contigs datasets. The prevalence of various ARG types is in line with literature [55]. Few genes were found in broilers fed MIX when compared to the C group (Fig. 5).

In particular, the frequency of chromosomal CfxA (beta-lactamase) and Erm(F) (erythromycin), genes were found to be lower in those broilers while the plasmid CepA (beta-lactamase), Tet(L) and Tet(M) (tetracycline) genes were absent in MIX broilers.The co-presence on mobile plasmids of erm(B), tet(M), tet(L) has been reported both in human and food enterococcal isolates [56, 57]. Those genes are commonly found in chickens [58, 59] and belong to Enterococci, and are associated with conjugative transposons, which explains their wide dissemination in the environment [57]. Moreover, spread of Beta lactamase genes in farms has been reported worldwide and this represents an issue of public health due to a possible route for transmission to consumers [60]. We observed a reduction of the incidence of several ARGs gene in broilers fed MIX (Fig. 5), and this finding was also observed in humans where a supplementation with a commercial probiotic preparation reduced the number of ARGs in the GI tract [61]. Moreover, enrichment in genes related to multidrug resistance and beta-lactamase antibiotic resistance were observed in *Faecalibacterium* pangenome from C broilers when compared to MIX.

## Conclusion

Dietary prebiotic/probiotic inclusion did not overall affect growth performance and histomorphological findings as well as the metabolome of the broilers, thus suggesting that these feeding strategies allow broilers to maintain required high growth standards. Functional differences and shift in the microbiome were highlighted due to the presence of the probiotics plus inulin in a synergic way. Several KEGG genes with potential beneficial effects on broiler health were associated with probiotic/prebiotic treatment and this finding should be considered in the perspective of developing novel, personalized strategies to promote feeding solutions for the poultry industry. The SCFAs profile displayed no significant differences, but the shift in the microbiota composition of few taxa that utilize SCFAs as an energy source indicate that the concomitant presence of probiotic and prebiotic stimulate the production of SCFAs that are used from other microbes in cross feeding relationships. In this way, the synergic effect of our feeding strategies promotes the microbiome selection at a strain-level. In particular, the distribution of *Faecalibacterium* strains was clearly related with the synergic effect of the probiotic/prebiotic treatment. A reduction in the prevalence of several ARGs was associated with the new strategy mediated by the strain level differences, thus potentially resulting in an improvement of animal welfare and safety and quality of poultry meat.

## Materials and methods

### Bacterial strains and probiotic preparation

The multi-strain probiotic preparation consisted of *Lactiplantibacillus plantarum* and *L. pentosus* from the Department of Agricultural, Forest and Food Sciences collection previously characterized for its in vitro probiotic ability [62]. Strains were cultured in MRS broth (VWR, Milan, Italy) overnight at 37 °C. One milliliter of fresh pure culture was subsequently cultured into 100 mL MRS broth and incubated for 24 h at 37 °C. Ten milliliter of fresh pure culture was subsequently cultured into 1000 mL MRS broth and incubated for 24 h at 37 °C. Cells were then harvested after centrifugation (14,000 × g, 30 min), washed twice with sterile Ringer solution and resuspended in Ringer. Viable cells per gram culture were determined by plating onto MRS Agar (VWR). Plates were incubated at 37 °C for 24 h under aerobic conditions. The two strains were combined to yield a total cell count of around 10^9^ CFU/g.

### Experimental design

A total of 160 1-day-old male broiler chicks (Ross 308) were randomly allotted to four dietary treatments, each consisting of 5 pens with 8 chicks per pen. Birds received regular vaccination against Newcastle disease, Marek disease, infectious bronchitis and coccidiosis at hatching. All the birds were maintained under the same environmental conditions (lighting schedule: 18 h light: 6 h dark; T: 32 °C during the first day, with reduction by 4 °C per week according to the age of the broilers until it reached 20 °C). A basal diet based on corn meal, corn gluten meal and soybean meal was formulated as previously described [63] and served as control group (C). Briefly, the nutritional composition for the first period (1-11d) basal diet was as follow: apparent metabolizable energy (AME): 12.55 MJ/kg; crude protein (CP): 230 g/kg; methionine (met): 5.60 g/kg; lysine (lys): 14.4 g/kg; Calcium (Ca): 9.60 g/kg and phosphorus (P): 4.80 g/kg. The nutritive value of the second period (12–32 days) basal diet was: AME: 12.97 MJ/kg; CP: 215 g/kg; met: 5.10 g/kg; lys: 12.9 g/kg; Ca: 8.70 g/kg and P: 4.30 g/kg. Four experimental treatments were then considered: (1) multi-strain probiotic (LABs, supplemented daily with water to yield 10^7^ CFU per broiler based on the water intake estimation/day), (2) multi-strain probiotic and 10% on top of inulin meal (MIX), and (3) 10% on top of inulin meal (I); (4) standard diet used as a control. The experimental period lasted 32 days.

### Growth performance

The live weight (LW) of the animals was recorded at an individual level at the beginning of the trial, on day 11 and at the end of the trial. The feed consumption was evaluated at pen level for the period 1–11 days and 12–32 days. Consequently, the feed conversion ratio (FCR) was determined at pen level for each growth period and for the overall experimental period. All the measurements were made on a pen basis using high precision electronic scales (Sartorius – Signum®).

### Faecal samplings

In order to collect fecal samples, all birds from each pen were housed in wire-mesh cages (100 × 50 cm width x length) for 30 min to collect fresh excreta samples. Fecal samples were collected at the beginning of the trial and after 5, 11 and 32 days. At each sampling point, fecal samples were then transferred with a sterile spatula in a 2 mL sterile Eppendorf tube and immediately stored at -80 °C.

### Slaughtering procedures and carcass traits

After a 10-h starvation period at 33 d of age a total of 40 bird selected on the basis of the average LW of the pen (two birds/pen per experimental diet;10 in total per treatment) were electrically stunned and slaughtered at a commercial abattoir. The plucked and eviscerated carcasses were obtained, and the head, neck, feet and abdominal fat were removed to obtain the chilled carcass.

### Samples collection

Blood samples were collected, at slaughtering, from the jugular vein of 12 randomly selected birds (3 animals per experimental diet) and were placed into serum-separating tubes, centrifuged at 2500 g for 10 min at 4 °C and the obtained serum was immediately frozen at -80 C.

Cecal content was immediately collected into sterile plastic tubes with a sterile spatula, immediately refrigerated (for a maximum of 2 h) and frozen at − 80 °C until DNA extraction and metabolomic analysis. Intestinal segment samples (approximately 5 cm in length) of duodenum, jejunum, ileum and caecum were excised. The collected segments of intestine were then flushed with 0.9% saline solution to remove all the content. The loop of the duodenum, the tract before Meckel’s diverticulum (jejunum), the tract before the ileocolic junction (ileum) and the apex of caecum. Samples of glandular stomach (proventriculus), liver, spleen, thymus and bursa of Fabricius were also collected.

### Blood parameters

Serum samples were analyzed for the following parameters: Albumine (g/dL), Alanine aminotransferase (UI/L), Aspartate aminotransferase (UI/L), Cholesterol, Triglycerides (mg/dL), Uric acid (mg/dL), Cholesterol HDL, Cholesterol LDL, Creatinine (mg/dL), Total Protein (g/dL), Phosphorous (mg/dl) Chlorine (mmol/L), Potassium (mmol/L), Magnesium (mEq/l), Iron (µg/dl), Alkaline Phosphatase (U/L), Calcium (mg/dl), Sodium (mmol/L). All the parameters were measured using an automated system photometer (I-Lab Aries Chemical Analyzer—Instrumentation Laboratory) [64]. These parameters were chosen since can indicate the functioning of different organ systems and help identify potential health issues particularly concerning liver function, kidney function, lipid metabolism, mineral imbalances, and overall nutritional status.

### Histomorphological investigations

Gut segments and organ samples were fixed in 10% buffered formalin solution for morphometric analysis (gut segments) and histopathological examination (gut and organ samples). Tissues were routinely embedded in paraffin wax blocks, sectioned at 5 μm thickness, mounted on glass slides and stained with Haematoxylin & Eosin (HE). The evaluated morphometric indices were villus height (Vh, from the tip of the villus to the crypt), crypt depth (Cd, from the base of the villus to the submucosa), and the villus height to crypt depth (Vh/Cd) ratio [65]. Morphometric analyses were performed on 10 well-oriented and intact villi and 10 crypts chosen from duodenum, jejunum, and ileum [66]. The observed histopathological findings were evaluated using a semi-quantitative scoring system as follows: absent (score = 0), mild (score = 1), moderate (score = 2) and severe (score = 3). Gut histopathological findings were separately assessed for mucosa (inflammatory infiltrates) and submucosa (inflammatory infiltrates and Gut-Associated Lymphoid Tissue [GALT] activation) for each segment. The total score of each gut segment was obtained by adding up the mucosa and submucosa scores, while the total score of each bird was obtained by adding up the duodenum, jejunum, ileum and caecum scores.

### Volatilome analysis

Volatiles from cecal samples [(0.0100 ± 0.005 g) precisely weighed in 10 mL headspace vials] were sampled by headspace (HS) solid-phase microextraction (SPME) with a divinylbenzene/carboxen/polydimethyl siloxane (DVB/CAR/PDMS) *d*_*f*_ 50/30 µm 2 cm length fiber (Supelco, Bellefonte, PA, USA). Sampling conditions were optimized in a previous study [67]. Internal standardization was included to normalize analytes responses and compensate for random errors inconsistencies. Sampling was at 70 °C for 30 min under constant agitation.

GC×GC analyses were performed on an Agilent 7890B GC unit (Agilent Technologies, Wilmington DE, USA) coupled to a Markes BenchTOF-Select™ mass spectrometer featuring Tandem Ionization™ (Markes International, Llantrisant, UK). The system was equipped with a two-stage KT 2004 loop-type thermal modulator (Zoex Corporation, Houston, TX) cooled with liquid nitrogen and controlled by Optimode v2.0 (SRA Intruments, Cernusco sul Naviglio, Milan, Italy).

Experimental conditions were optimized for VOCs profiling, parameters are reported in the reference study by Squara et al. [68] .

The data processing workflow was by computing peak and peak-region features from untargeted (unknowns) and targeted components located over the 2D space. By this approach, named *UT* fingerprinting [69], the targeting (i.e., identification) of analytes was by spectral similarity DMF ≥ 900 and RMF ≥ 950 and linear retention index (*I*^*T*^) tolerance ± 5 units.

For the purpose of this study, SCFAs normalized % responses (i.e., 2D peak chromatographic volume normalized over total response), were collected and observed as a function of the treatment.

### DNA extraction and metagenomic sequencing

Nucleic acid was extracted by fecal samples at each sampling point and from cecal content. Total DNA from the samples was extracted using the RNeasy Power Microbiome KIT (Qiagen. Milan. Italy) following the manufacturer’s instructions. One microliter of RNase (Illumina Inc. San Diego. CA) was added to digest RNA in the DNA samples with an incubation of 1 h at 37 °C.

DNA directly extracted from fecal and cecal samples was used to assess the microbiota by the amplification of the V3-V4 region of the 16S rRNA by using the primers 16SF 5’-CCTACGGGNGGCWGCAG-3’ and 16SR 5’-GACTACHVGGGTATCTAATCC-3’ following the procedure already reported [70]. PCR products were purified, tagged, quantified and pooled according to the Illumina16S sequencing library preparation protocol. Sequencing was performed with a MiSeq Illumina instrument with V2 chemistry and generated 250 bp paired-end reads according to the manufacturer’s instructions. Whole metagenomics (150 bp paired-end reads) was performed only for cecal content samples on a NovaSeq Illumina platform by the Genewiz company (Leipzig, Germany).

### Bioinformatics analysis

16 S rRNA amplicon datasets were analyzed by using QIIME 2 software, as recently reported [71]. Briefly Amplicon Sequence Variants (ASVs) were obtained from denoised reads by the DADA2 algorithm [72] and mapped against the Greengenes 13.5v 16 S rRNA gene database by using QIIME2. Taxonomy of the ASVs table was manually checked by BLASTn for consistence and ASVs table was rarefied at the lowest number of sequence/samples.

The whole metagenome data analysis was performed as follows. The obtained reads were mapped to the draft genome of *Gallus gallus* (GRCg6a) by Bowtie2 (v.2.4.4) in end-to-end sensitive mode [73]. Non host clean reads were quality filtered with Solexa QA + + software (v.3.1.7.2) [74] and sequences less than 60 bp and dereplicated sequences were filtered by Prinseq (v.0.20.4) [75]. A total of 68.51 Gb sequences, with an average of 5.81 Gb per sample were then used for downstream analysis. Reads were assembled with MetaSPAdes (version 3.11.0) [76], and the quality of the contigs was checked with QUAST (v.5.0.2) [77]. Contigs shorter than 1,000 nt were discarded. Reads assembled into 272.401 contigs longer than 1000 bp. The total contig length per sample in average was 75 Mb with an average N50 length of 5.000 bp.

From each contig, a total of 430.982 genes were predicted by using MetaGeneMark (v. 3.26). The pipeline used to obtain the gene catalogs is described elsewhere [78]. Concatenated genes were aligned against the NCBI-NR database and clean reads were mapped back by using Bowtie2 to the annotated gene catalogue to enable semiquantitative analysis. Reads mapped to each gene were then used for functional analysis against KEGG database by MEGAN6 software [79]. KEGG gene count table was internally normalized in MEGAN by checking the “use normalized count” option. For the same set of metagenomes, we used MetaPhlAn3 [80] to estimate the relative abundance of taxa at species level. Metagenome-assembled genomes (MAGs) were extract from contigs with MetaBat2 (v.2.12.1) [81] and quality was evaluate with CheckM [82]. Only 52 high-quality MAGs with > 90% completeness, < 5% contamination were then used for the downstream analysis according to the recent guidelines [83]. Taxonomic assignment was performed with Focus [84] and confirmed by using the assign taxonomic label script from PhyloPhlAn3 [85]. Phylogenetic tree was then inferred with PhyloPhlAn3 and visualized in iTOL6 (https://itol.embl.de/). Pangenome calculation and marker gene identification was achieved by Prokka [86] and Roary [87] using the UniRef90 database of UniProt. In order to find the acquired antimicrobial resistance genes in the assembled metagenomes, *AMRFinderPlus* was used [54]. Only genes with coverage and identity > 98% and identity were selected. *SourceFinder* was then used for identification of chromosomal, plasmid, and bacteriophage sequences [88].

### Statistical analysis

The statistical analysis regarding bird performance and histomorphology was performed using the IBM SPSS Statistics package (IBM Corp. Released 2011. IBM SPSS Statistics for Windows, Version 20.0. Armonk, NY: IBM Corp.). Normality of the data distribution and homogeneity of variances were assessed using the Shapiro–Wilk test and the Levene test, respectively. The experimental unit was the pen for growth performance, while the individual bird was used for the blood traits, slaughter performance and histological findings, taking the pen as a random effect. The collected data were analysed according to the General Linear Model procedure with the treatment as the principal effect and the pen as a random effect. Multiple comparisons were performed using Tukey’s HSD test, when variances among groups were homogeneous, and Games-Howell test, when variances were not homogeneous. Intestinal morphometric indices were analyzed by fitting a General Linear Model. The model allowed the morphometric indices (Vh, Cd, and Vh/Cd, separately) to depend on three fixed factors (diet, intestinal segment, and interaction between diet and intestinal segment). The interactions between the levels of the fixed factors were evaluated by pairwise comparisons. Statistical analysis was performed by procedure “General Linear Models > Univariate”. Histopathological scores were analyzed by Kruskal-Wallis test (post hoc test: Dunn’s Multiple Comparison test). Significance was declared at *P* < 0.05. A statistical trend was considered for *P* < 0.10. Results were expressed as mean and pooled standard error of the mean (SEM).

For the metataxonomic dataset, alpha diversity indices were calculated using the diversity function of QIIME2. A Generalized Linear Model was used in order to test the importance of continuous or discrete variables available for the birds (sampling time and diet) on the relative frequency of bacterial genus or family. Diversity index, metabolome data and microbiota data were analyzed using the Kruskal-Wallis test to assess differences between the feeding diet and visualized by boxplot. P values were adjusted for multiple testing using the Benjamini-Hochberg procedure, which assesses the false-discovery rate (FDR). Determination of differentially abundant genes was then conducted using the Bioconductor DESeq2 package [89] in the statistical environment R. The genes presence/absence table obtained from the reconstructed MAGs was used to calculate the distance matrix on Bray Curtis’s distance by the *vegdist* function in package vegan of R.

### Electronic supplementary material

Below is the link to the electronic supplementary material.

Supplementary Material 1: Additional file 1 — Table S1. Effects of diet, intestinal segment and interaction between diet and intestinal segment on the intestinal morphometric indices of the broiler chickens fed with dietary probiotic inclusion. Table S2. Least square means of intestinal morphometric indices in broilers in relation to diet and intestinal segment. Table S3. Effects of diet and sampling time and interaction between diet and sampling time intestinal on the faecal ASVs of the broiler chickens fed with dietary probiotic inclusion.

Supplementary Material 2. Additional file 2 — Table S4. List of KEGG Orthology (KO) genes differentially (FRD < 0.05) detected in the metagenome between, broilers fed with control (C) and Inulin plus probiotics (MIX). Positive log 2 fold changes indicate genes most abundant in C samples. Table S5. List of KEGG Orthology (KO) genes differentially (FRD < 0.05) detected in the metagenome, between broilers fed with control (C) or Inulin (I). Positive log 2 fold changes indicate genes most abundant in C samples. Table S6. List of KEGG Orthology (KO) genes differentially (FRD < 0.05) detected in the metagenome, between broilers fed with control (C) or and probiotics (LABs). Positive log 2 fold changes indicate genes most abundant in LABs samples.

## Data Availability

Sequences data were deposited on NCBI database under the bioproject number PRJNA836733.
