# Supplementary material for: Lactiplantibacillus plantarum, lactiplantibacillus pentosus and inulin meal inclusion boost the metagenomic function of broiler chickens
Source: Anim Microbiome. 2023 Aug 3;5:36. doi: 10.1186/s42523-023-00257-5 (PMC10399007; doi:10.1186/s42523-023-00257-5)
Supplement: Supplementary file 1 — Supplementary Material 1: Additional file 1 — Table S1. Effects of diet, intestinal segment and interaction between diet and intestinal segment on the intestinal morphometric indices of the broiler chickens fed with dietary probiotic inclusion. Table S2. Least square means of intestinal morphometric indices in broilers in relation to diet and intestinal segment. Table S3. Effects of diet and sampling time and interaction between diet and sampling time intestinal on the faecal ASVs of the broiler chickens fed with dietary probiotic inclusion. [file 42523_2023_257_MOESM1_ESM.docx]

**Table S1.** Effects of diet, intestinal segment and interaction between diet and intestinal segment on the intestinal morphometric indices of the broiler chickens fed with dietary probiotic inclusion.

| **Index** | **Fixed effect** | **d.f.^3^** | **F** | ***P*-value^4^** |
| --- | --- | --- | --- | --- |
| Vh (mm) | Diet^1^ | 3 | 0.321 | 0.810 |
|  | Intestinal segment^2^ | 2 | 175.949 | < 0.001 |
|  | Diet × Intestinal segment | 6 | 2.701 | 0.019 |
| Cd (mm) | Diet | 3 | 2.043 | 0.114 |
|  | Intestinal segment | 2 | 4.325 | 0.016 |
|  | Diet × Intestinal segment | 6 | 0.423 | 0.861 |
| Vh/Cd (mm/mm) | Diet | 3 | 0.335 | 0.800 |
|  | Intestinal segment | 2 | 149.957 | < 0.001 |
|  | Diet × Intestinal segment | 6 | 4.225 | 0.001 |

^1^Four dietary treatments: C = control; LABs *=* *Lactiplantibacillus plantarum + L. pentosus*; I = inulin; MIX = inulin + *Lactiplantibacillus plantarum + L. pentosus Lactobacillus*. ^2^Three intestinal segments: duodenum, jejunum and ileum. ^3^Degrees of freedom. ^4^Statistical significance: P < 0.05. Vh, villus height; Cd, crypt depth; Vh/Cd, villus height to crypt depth ratio.

**Table S2.** Least square means of intestinal morphometric indices in broilers in relation to diet and intestinal segment.

| **Index** | **Fixed effect** | **Effect levels** | **Least square mean^1^** | **SEM** |
| --- | --- | --- | --- | --- |
| Vh (mm) | Diet^2^ | C2 | 2.33 | 0.09 |
|  |  | CL | 2.33 |  |
|  |  | CI | 2.31 |  |
|  |  | CIL | 2.22 |  |
|  | Intestinal segment^3^ | DU | 3.49^a^ | 0.08 |
|  |  | JE | 1.97^b^ |  |
|  |  | IL | 1.44^c^ |  |
| Cd (mm) | Diet | C2 | 0.19 | 0.01 |
|  |  | CL | 0.19 |  |
|  |  | CI | 0.19 |  |
|  |  | CIL | 0.18 |  |
|  | Intestinal segment | DU | 0.20^a^ | 0.01 |
|  |  | JE | 0.19^ab^ |  |
|  |  | IL | 0.18^b^ |  |
| Vh/Cd (mm/mm) | Diet | C2 | 12.12 | 0.49 |
|  |  | CL | 11.83 |  |
|  |  | CI | 12.15 |  |
|  |  | CIL | 12.52 |  |
|  | Intestinal segment | DU | 17.99^a^ | 0.42 |
|  |  | JE | 10.37^b^ |  |
|  |  | IL | 8.10^c^ |  |

^1^Means with different superscript letters (a, b, c) within the same column per fixed effect (i.e. diet, intestinal segment) differ significantly (P < 0.05). ^2^C = control; LABs *=* *Lactiplantibacillus plantarum + L. pentosus*; I = inulin; MIX = inulin + *Lactiplantibacillus plantarum + L. pentosus Lactobacillus*. ^3^DU = duodenum; JE = jejunum; IL = ileum. Vh, villus height; Cd, crypt depth; Vh/Cd, villus height to crypt depth ratio. SEM: pooled standard error of the mean.

**Table S3.** Effects of diet and sampling time and interaction between diet and sampling time intestinal on the faecal ASVs of the broiler chickens fed with dietary probiotic inclusion.

| **Fixed effect^1^** | **ASVs** | **Df^2^** | **F^3^** | ***P*-value** |
| --- | --- | --- | --- | --- |
| Diet x Time | *Aerococcus* | 6 | 2,41 | 0,036 |
|  | *Bacteroides* | 6 | 4,682 | 0,001 |
|  | *Helicobacter* | 6 | 2,882 | 0,015 |
|  | *Lactobacillus* | 6 | 11,418 | < 0.001 |
|  | *Leuconostocaceae* | 6 | 3,297 | 0,007 |
|  | *Parabacteroides* | 6 | 3,487 | 0,005 |
|  | *Peptococcus* | 6 | 2,618 | 0,025 |
|  | *Phascolarctobacterium* | 6 | 2,501 | 0,031 |
|  | *Rikenellaceae* | 6 | 3,825 | 0,003 |
|  | *Ruminococcus* | 6 | 2,393 | 0,038 |
|  | *Sutterella* | 6 | 3,12 | 0,01 |
|  | *Turicibacter* | 6 | 3,467 | 0,005 |
|  | *Veillonella* | 6 | 24,436 | < 0.001 |
| Time | *Aerococcaceae* | 3 | 3,028 | 0,036 |
|  | *Aerococcus* | 3 | 4,578 | 0,006 |
|  | *Akkermansia* | 3 | 38,586 | < 0.001 |
|  | *Alloiococcus* | 3 | 3,189 | 0,029 |
|  | *Citrobacter* | 3 | 29,01 | < 0.001 |
|  | *Collinsella* | 3 | 40,123 | < 0.001 |
|  | *Delftia* | 3 | 20,042 | < 0.001 |
|  | *Enterococcus* | 3 | 33,765 | < 0.001 |
|  | *Helicobacter* | 3 | 4,136 | 0,01 |
|  | *Klebsiella* | 3 | 31,854 | < 0.001 |
|  | *Lactobacillus* | 3 | 22,088 | < 0.001 |
|  | *Leuconostocaceae* | 3 | 5,382 | 0,002 |
|  | *Parabacteroides* | 3 | 5,607 | 0,002 |
|  | *Peptococcus* | 3 | 4,001 | 0,011 |
|  | *Phascolarctobacterium* | 3 | 3,269 | 0,027 |
|  | *Proteus* | 3 | 2,685 | 0,054 |
|  | *Rikenellaceae* | 3 | 5,783 | 0,001 |
|  | *Ruminococcus* | 3 | 3,056 | 0,034 |
|  | *Sutterella* | 3 | 3,569 | 0,019 |
|  | *Turicibacter* | 3 | 4,982 | 0,004 |
| Diet | *Sutterella* | 3 | 3,028 | 0,036 |
|  | *Turicibacter* | 3 | 2,871 | 0,043 |

^1^Four dietary treatments: C = control; LABs *=* *Lactiplantibacillus plantarum + L. pentosus*; I = inulin; MIX = inulin + *Lactiplantibacillus plantarum + L. pentosus Lactobacillus*. ^2^Degrees of freedom. ^3^Statistical significance: P < 0.05.
